# Hyaluronan Binding Motifs of USP17 and SDS3 Exhibit Anti-Tumor Activity

**DOI:** 10.1371/journal.pone.0037772

**Published:** 2012-05-25

**Authors:** Suresh Ramakrishna, Bharathi Suresh, Su-Mi Bae, Woong-Shick Ahn, Key-Hwan Lim, Kwang-Hyun Baek

**Affiliations:** 1 Department of Biomedical Science, CHA University, CHA General Hospital, Gyeonggi-Do, Republic of Korea; 2 Catholic Research Institute of Medical Science, The Catholic University of Korea College of Medicine, Seoul, Republic of Korea; University of Navarra, Spain

## Abstract

**Background:**

We previously reported that the USP17 deubiquitinating enzyme having hyaluronan binding motifs (HABMs) interacts with human SDS3 (suppressor of defective silencing 3) and specifically deubiquitinates Lys-63 branched polyubiquitination of SDS3 resulting in negative regulation of histone deacetylase (HDAC) activity in cancer cells. Furthermore, USP17 and SDS3 mutually interact with each other to block cell proliferation in HeLa cells but the mechanism for this inhibition in cell proliferation is not known. We wished to investigate whether the HABMs of USP17 were responsible for tumor suppression activity.

**Methodology/Principal Findings:**

Similarly to USP17, we have identified that SDS3 also has three consecutive HABMs and shows direct binding with hyaluronan (HA) using cetylpyridinium chloride (CPC) assay. Additionally, HA oligosaccharides (6-18 sugar units) competitively block binding of endogenous HA polymer to HA binding proteins. Thus, administration of HA oligosaccharides antagonizes the interaction between HA and USP17 or SDS3. Interestingly, HABMs deleted USP17 showed lesser interaction with SDS3 but retain its deubiquitinating activity towards SDS3. The deletion of HABMs of USP17 could not alter its functional regulation on SDS3-associated HDAC activity. Furthermore, to explore whether HABMs in USP17 and SDS3 are responsible for the inhibition of cell proliferation, we investigated the effect of USP17 and SDS3-lacking HABMs on cell proliferation by soft agar, apoptosis, cell migration and cell proliferation assays.

**Conclusions:**

Our results have demonstrated that these HABMs in USP17 and its substrate SDS3 are mainly involved in the inhibition of anchorage-independent tumor growth.

## Introduction

The homeostasis of a number of cellular proteins is regulated by many molecular events that help to define the status and fate of physiological processes. Such regulatory events include phosphorylation, dephosphorylation, acetylation, deacetylation, and ubiquitination. The process of ubiquitination is a well-established event involving a complex yet organized milieu of ubiquitin-activating enzymes (E1), ubiquitin-conjugating enzymes (E2), and ubiquitin ligase (E3) enzymes [1], [2], [3], [4]. Deubiquitinating enzymes (DUBs) are a large family of proteases, which counterbalance the ubiquitination process by removing ubiquitin molecules from its substrates [3], [5], [6]. Most DUBs are cysteine proteases and consist of five known families: the ubiquitin C-terminal hydrolases (UCH), the ubiquitin-specific processing proteases (USP), Jab1/Pab1/MPN-domain containing metallo-enzymes (JAMM), Otu-domain ubiquitin aldehyde-binding proteins (OTU), and Ataxin-3/Josephin [3], [4], [7].

USP17 was previously identified as a human orthologue DUB-3 that is regulated by IL-4 and IL-6 cytokines [8]. Several evidences have proved that USP17 acts as a key regulator of cell proliferation and survival [8], [9], [10], [11]. More recently, chemokine regulation of USP17 has been noticed in both elongated and amoeboid cell motility. Depletion of endogenous expression of USP17 inhibits normal cytoskeletal rearrangements and chemokine-induced membrane localization of Rho GTPases [12]. In a previous study, we showed that USP17 possesses two hyaluronan binding motifs (HABMs) in its C-terminus region and interacts with hyaluronan (HA) to regulate cell viability [13]. These HABMs are termed B-X7-B motifs, where B refers to two flanking basic amino acid residues (either arginine or lysine) and X is a non-acidic amino acid [14]. However, the biological significance of these HABMs in USP17 is not yet fully understood. In a recent study, we reported that USP17 specifically deubiquitinates K63-linked ubiquitination of SDS3 and negatively regulates SDS3-associated HDAC activity in cancer cells [11].

HA is a negatively charged natural polysaccharide with a high molecular weight, and is a common component of synovial fluid and extracellular matrix. HA is responsible for various functions within the extracellular matrix such as cell growth, adhesion, differentiation, migration, proliferation, tumor formation, metastasis and invasion [15], [16]. In addition to its role in the extracellular matrix, several evidences have proved that HA is also present in the rough endoplasmic reticulum (ER) membranes, plasma membranes, cytoplasm and nuclei of cells in a number of tissues *in vivo* [17], [18], [19], [20]. Several intracellular HA-binding proteins have been reported to be involved in the regulation of cell cycle or gene transcription [21], [22], [23], [24], [25], [26]. Additionally, an intracellular form of the HA receptor, RHAMM, has been shown to be responsible for regulating extracellular-regulated kinase activity [24]. HA is found to accumulate intracellularly in the perinuclear region of aortic smooth muscle cells during premitotic and mitotic stages [27]. This HA is found to aggregate at the mitotic spindle and facilitate the process of nuclei separation and subsequent cell division [20]. HA is closely associated with the nuclei forming a cable structure that extends beyond the original location of the cell membranes, suggesting that these HA cables are initiated either by perinuclear or ER membranes [20]. The accumulation of HA cables is elevated during ER stress in smooth muscle cells [28]. In addition, HA cables serve as a distress signal during inflammatory stimulus leading to monocyte activation and also are capable of binding to leukocytes [28]. However, several functions of intracellular HA are predicted during various cellular processes, and the source of these intracellular HAs is not clearly understood yet.

Perturbation of endogenous HA interaction in tumor cells inhibits tumor growth, invasion and metastasis [29], [30], [31], [32], [33]. In addition, HA oligosaccharides have been shown to inhibit tumor growth and induce apoptosis. Additionally, a synthetic peptide containing at least three HABMs showed reduced tumor growth and induced apoptosis [34], [35]. HA oligosaccharides competitively block binding of endogenous HA polymer to CD44, consequently giving rise to attenuated signaling. HA oligosaccharides also induce apoptosis and stimulate caspase-3 activity under anchorage-independent conditions [36], which was similar to the action of USP17-containing HABMs transfected into cervical adenocarcinoma cells causing apoptosis [11], [13]. Thus, elucidating the role of HABMs in USP17 on cell proliferation and its implications on regulation of apoptosis in tumor cells is highly desired.

## Results and Discussion

### Hyaluronan-binding Properties of USP17

**Figure 1:**
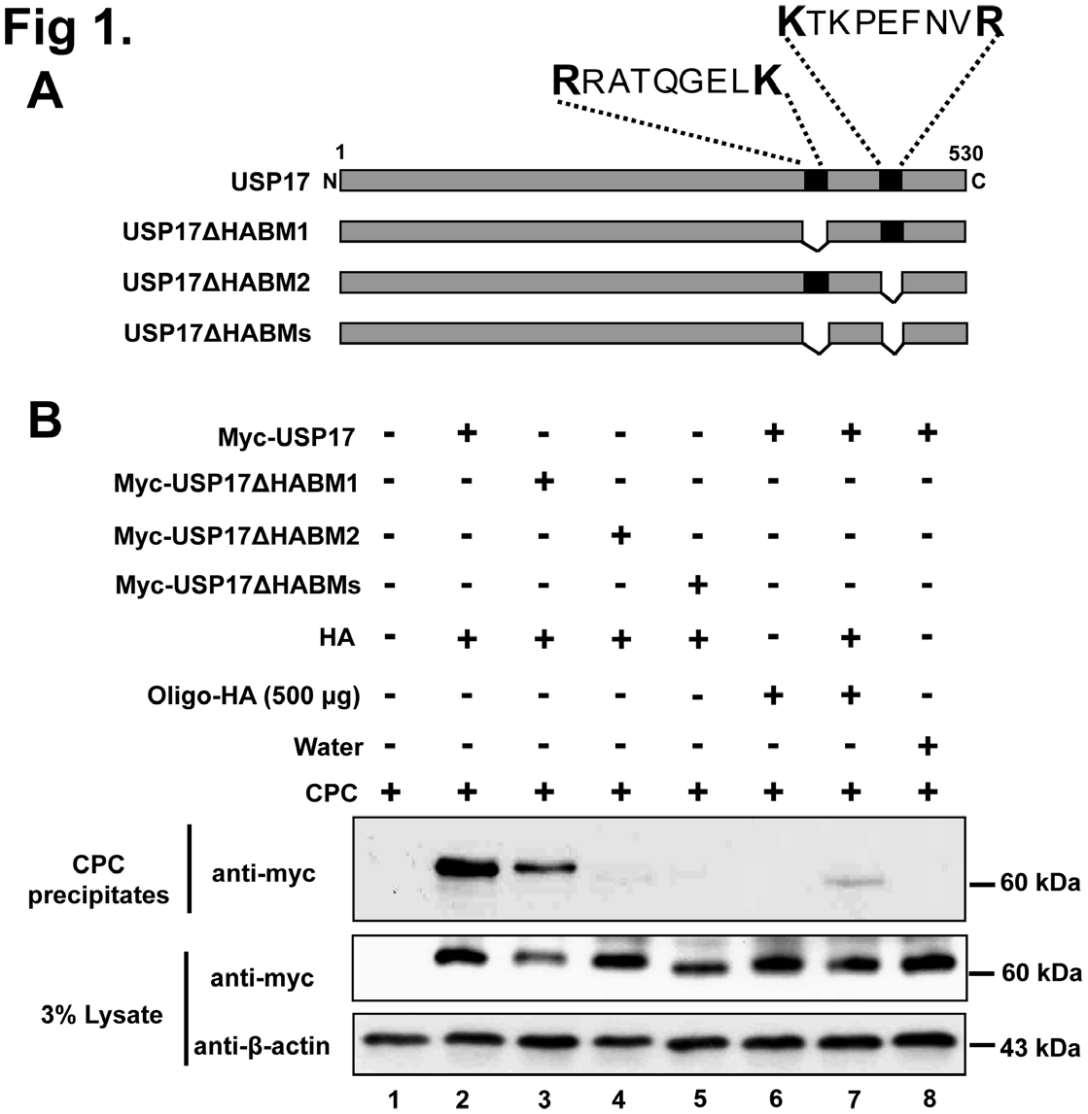
Hyaluronan-binding activity of USP17. **A**) Schematic representation of HABM-deleted constructs of USP17. **B**) HA binding to HABM-deleted constructs of USP17. Cell lysate aliquots from 293 T cells transfected with different USP17 deletion constructs were incubated with HA and HA oligosaccharides and subjected to CPC precipitation. Western blot analysis was performed using an anti-myc antibody. The lower panel contains an aliquot of whole cell lysate without addition of CPC or HA. **C**) Binding of USP17 to HA oligosaccharides. Cell lysate aliquots from 293 T cells transfected with USP17 were incubated with different concentration of HA oligosaccharides and subjected to CPC precipitation followed by Western blot analysis with an anti-myc antibody. **D**) Cell lysate aliquots from 293 T cells transfected with USP17 and HABMs-deleted USP17 were incubated with high concentration of HA oligosaccharides (2000 µg) and performed CPC precipitation followed by Western blot analysis. **E**) Cell lysate aliquots from 293 T cells transfected with USP17 were incubated with 20 µg of each USP17 peptide or together in the presence of HA and performed CPC precipitation followed by Western blot analysis.

HA is enriched in pericellular matrices surrounding proliferating and migrating cells. Elevated HA biosynthesis is a common feature in many types of human cancers, and its constitutive interaction with tumor cells has a major influence on tumor growth and metastasis, promoting anchorage-independent growth and invasiveness in animal models [37], [38]. In a previous study, we showed that USP17 possesses two HABMs [HABM1 (RRATQGELKR) and HABM2 (KTKPEFNVR)] in its C-terminal region that is located at amino acids 401 to 410, and 445 to 453, respectively [13]. To find out which HABM is critical for HA-binding of USP17, we generated a combination of HABM-deletion constructs of USP17 for further investigation (**Figure 1A**). We examined the HA-binding properties of USP17 by using cetylpyridinium chloride (CPC) to specifically precipitate glycosaminoglycans. CPC is a cationic detergent that has a tendency to bind and precipitate anionic glycosaminoglycans resulting in co-precipitation of binding partners [39]. Thus, the CPC precipitation assay showed that the deletion of the second HABM results in a significant reduction of HA binding (**Figure 1B** **, lane 4**), and both HABM-deletion constructs showed a marked reduction in HA binding (**Figure 1B** **, lane 5**).

HA oligosaccharides inhibit growth of several types of tumors *in vivo* [40]. These HA oligosaccharides competitively block binding of endogenous HA polymer to HA-binding proteins [41]. Therefore, we wished to investigate the effect of HA oligosaccharides on HA-binding of USP17. To investigate whether HA oligosaccharides bind with USP17, we examined the minimum concentration of HA oligosaccharides required to precipitate USP17 using the CPC assay. Due to the small size of HA oligosaccharides, it was unlikely to observe any precipitation using the CPC assay, but with a high amount of HA oligosaccharides ranging between 1000 and 2000 µg/ml, precipitation of USP17 could be detected (**Figure 1C**). In contrast, the same high amount of oligosaccharides (2000 µg/ml) could not precipitate both HABMs deleted USP17 (**Figure 1D** **, lane 4**), indicating the specific binding between USP17 and HA oligosaccharides. Thus, our result shows that HA oligosaccharides have binding capacity for USP17. We next examined the ability of HA oligosaccharides to antagonize the interaction between USP17 and HA. Interestingly, we observed a significant reduction in USP17 binding with polymeric HA in the presence of HA oligosaccharides compared to that in the absence of HA oligosaccharides (**Figure 1B** **, lane 7**). As a negative control, distilled water was added instead of HA (**Figure 1B** **, lane 8**). This result signifies that HA oligosaccharides show antagonistic effect on USP17 binding with polymeric HA. In order to address the specificity of HABMs, we synthesized the peptides mimicking HABMs of USP17 and performed CPC assay (**Figure 1E**). The blocking effect of peptides on the interaction between HA and USP17 was shown even though it was not as strong as HA oligosaccharides (**Figure 1E** **, lanes 3-5**). Thus, our result indicates that HA oligosaccharides have specific binding capacity for HABMs of USP17.

### SDS3 Binds with HA

Mouse SDS3 (mSDS3) is a key component of the mSin3-HDAC complex, sharing physical and functional properties of its yeast ortholog and shares 99.6% identity with human SDS3. The impact of mSDS3 haploinsufficiency on cancer development along with p53-dependent checkpoint control reveals that loss of one mSDS3 allele accelerates tumor onset and increased tumor burden in p53 null background [42], [43].

**Figure 2:**
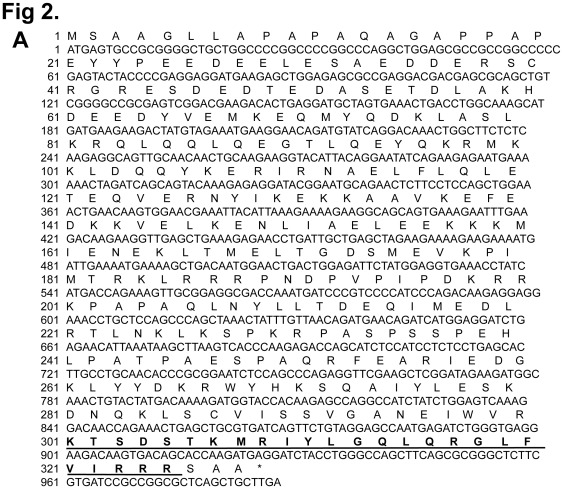
Binding of SDS3 to HA . **A**) Identification of three overlapping HABMs in SDS3 nucleotide and deduced amino acid sequence. HABMs are represented in bold face and underlined (301-325 aa). **B**) Comparison and alignment of SDS3 amino acid sequence and BH-P. **C**) SDS3 binds with HA. CPC precipitation assay was performed on 293 T cells transfected with USP17, SDS3 and SDS3/USP17-shRNA. Proteins precipitated with HA were subjected to Western blot analysis with either an anti-myc or an anti-Flag antibody. **D**) USP17 and SDS3 directly bind with HA. CPC precipitation assay was performed on purified GST-tagged USP17 or His-tagged SDS3 proteins separately. Western blot analysis with either an anti-GST or an anti-His antibody was performed.

In a recent study, human SDS3 was identified as a potential binding partner for USP17 by MALDI-TOF analysis [11]. The functional interaction between USP17 and SDS3 was responsible for inducing apoptosis through negative regulation of HDAC activity. Thus, USP17 and SDS3 mutually interact with each other to affect cell viability of cancer cells. The fact that SDS3 alone has an inhibitory effect on cell proliferation led us to speculate that SDS3 may also have some motifs within its sequence that are responsible for blocking cell proliferation. An examination of the deduced amino acid sequence of SDS3 revealed three consecutive overlapping HABMs (301-KTSDSTKMRIYLGQLQRGLFVIRRR-325) in its C-terminus (**Figure 2A**). More interestingly, it has been reported that a synthetic peptide having at least three HABMs showed reduced tumor growth and induced apoptosis [35]. Later, it has been shown that a 42-amino acid peptide (designated as BH-P) containing several HABMs from human brain HA-binding protein inhibits tumor growth and induces apoptosis [34]. These results indicate that the HABMs are responsible for the anti-tumor effect. We compared and aligned the amino acid sequences of the HA-binding regions of SDS3 to synthetic peptide BH-P (**Figure 2B**). We investigated whether SDS3 binds to HA, by performing the CPC assay. As a positive control, USP17 binding to HA was taken into consideration for the CPC assay. SDS3 was readily detected in the CPC precipitates after preincubation with exogenous HA, in contrast to water, which was taken as a negative control (**Figure 2C**). Next, we wished to examine whether the HA binding ability of SDS3 was acquired due to its interaction with USP17. For this purpose, we investigated the HA binding ability of SDS3 in the presence of depletion of endogenous USP17. Our result suggests that SDS3 binds to HA and is independent to its interaction with USP17 (**Figure 2C** **lane 4**). To investigate the direct interaction between HA and USP17 or SDS3, we incubated purified His-tagged SDS3 and GST-tagged USP17 proteins separately with polymeric HA and performed the CPC assay (**Figure 2D**). The CPC precipitation assay showed that USP17 and SDS3 directly bind with HA.

### Hyaluronan-binding Properties of SDS3

**Figure 3:**
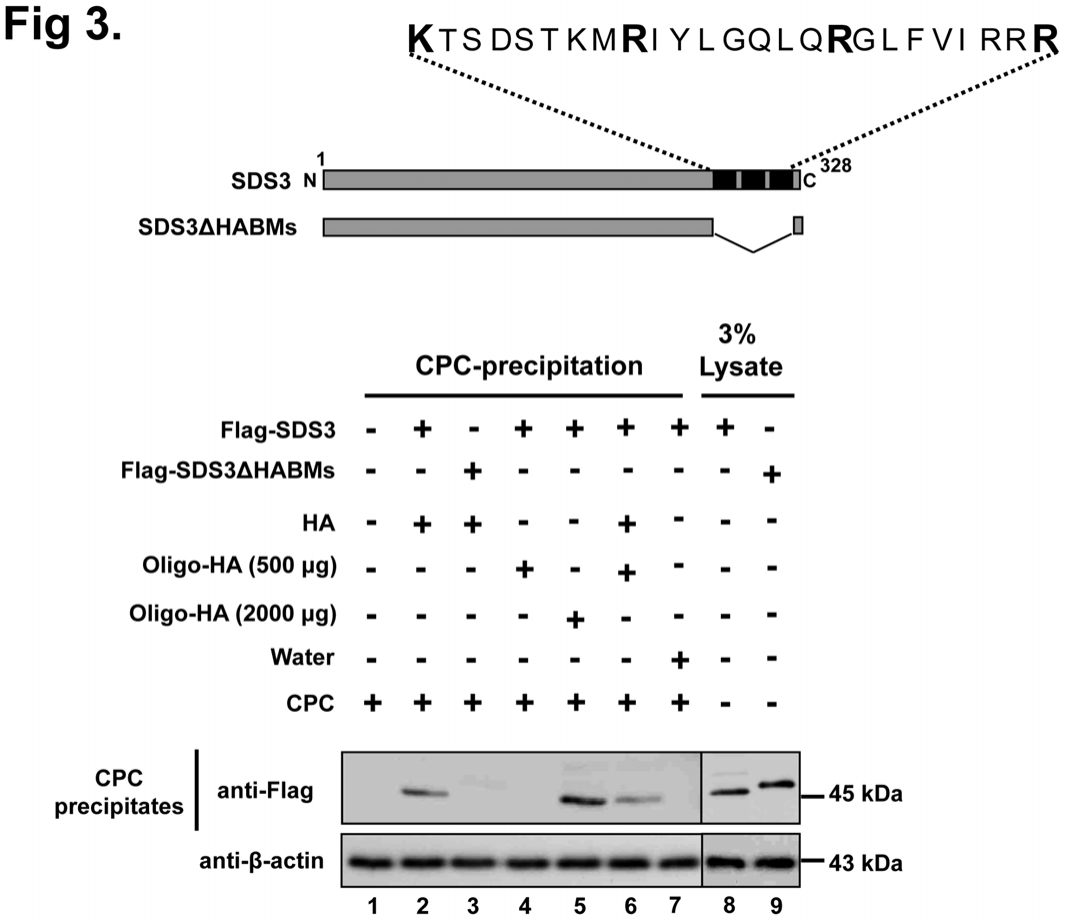
Hyaluronan binding activity of SDS3. Cell lysate aliquots from 293 T cells transfected with SDS3 and HABM-deleted construct of SDS3 were incubated with HA and different concentration of HA oligosaccharides, and subjected to CPC precipitation followed by Western blot analysis with an anti-Flag antibody.

Identification of three consecutive HABMs in SDS3 led us to validate HA-binding properties of SDS3 by deleting three consecutive HABMs (301-KTSDSTKMRIYLGQLQRGLFVIRRR-325) and we analyzed the HA-binding ability of SDS3 by CPC assay. From CPC assay, we found that the HA-binding ability of SDS3 was greatly reduced in the absence of the HABMs (**Figure 3** **, lane 3**). These results suggest that the HABMs are critical for HA-binding of SDS3. We further confirmed the binding tendency of SDS3 with HA oligosaccharides at different concentrations and found precipitation of SDS3 only at a high concentration (**Figure 3** **, lane 5**). The effect of HA oligosaccharides at a concentration of 500 µg/ml showed an antagonistic effect on SDS3 binding with polymeric HA (**Figure 3** **, lane 6**). Overall, the data indicate that the interaction between polymeric HA and SDS3 was competitively inhibited by low molecular weight HA oligosaccharides, suggesting that HABMs in SDS3 are essential for HA-binding and imply a distinct HA-binding region on SDS3.

### HABM-deletion Construct of USP17 has Less Binding Affinity for SDS3 but Retain its Catalytic Activity on SDS3 Protein

**Figure 4:**
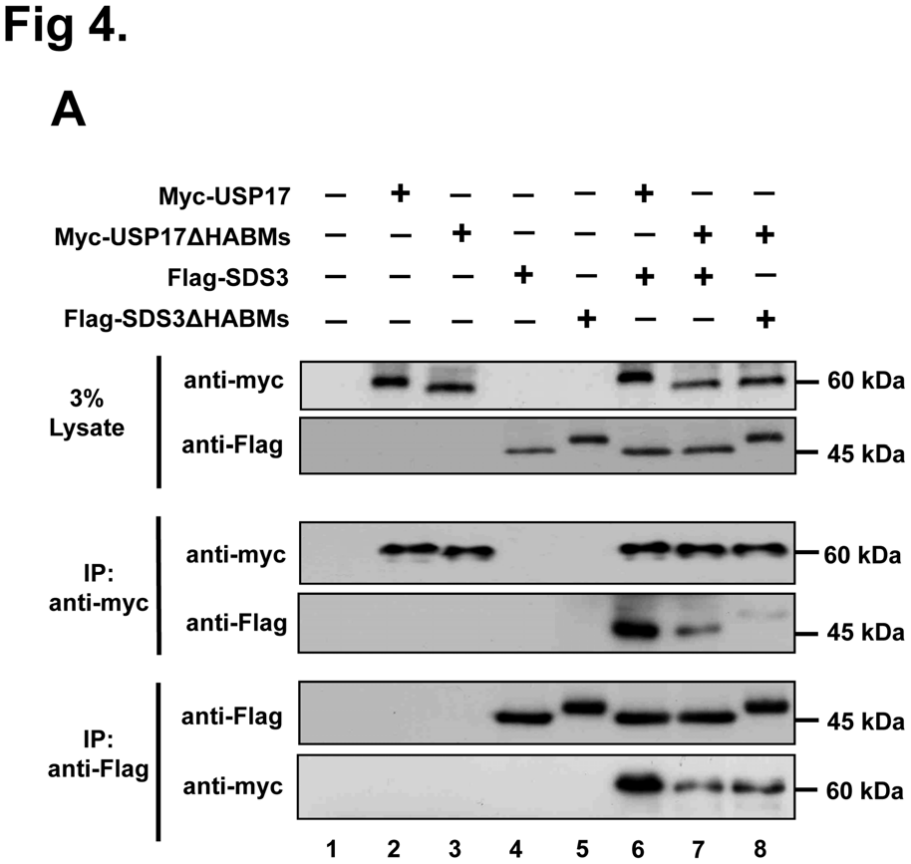
HABMs deleted USP17 deubiquitinates SDS3. **A**) Interaction between HABMs deleted USP17 and SDS3. **B**) Interaction between USP17ΔHABM1 or USP17ΔHABM2 and SDS3. Interaction was confirmed by co-immunoprecipitation with either an anti-myc antibody or an anti-Flag antibody. **C**) Deubiquitination of SDS3 by HABMs deleted USP17. 293 T cells were transfected with the respective expression constructs. A deubiquitinating assay was performed by co-immunoprecipitation with an anti-Flag antibody and immunoblotting using an anti-HA antibody.

We inquired whether deletion of HABMs from USP17 affects its interaction and deubiquitinating activity on SDS3. We therefore conducted a binding assay between HABM- deletion construct of USP17 and SDS3. Interestingly, HABM-deletion construct of USP17 showed significantly lesser interaction with SDS3 when compared with the full length USP17 (**Figure 4A** **, lanes 6 and 7**). In addition, HABM-deletion construct of USP17 and HABM-deletion construct of SDS3 showed weaker interaction (**Figure 4A** **, lane 8**). Furthermore, we investigated the interaction between SDS3 and first HABM deleted USP17 or second HABM deleted USP17. The result showed that the second HABM deleted USP17 has weak binding intensity than the first HABM deleted USP17 with the full length SDS3 (**Figure 4B**). However, the overlapped arrangement of HABMs within SDS3 was difficult in characterizing the binding intensity of each HABM of SDS3 with the full length USP17.

Then, we wished to investigate the deubiquitinating activity of HABM-deletion construct of USP17 on SDS3. The HABM-deletion construct of USP17 could deubiquitinate SDS3 which was similar to the activity of the full length USP17 (**Figure 4C** **, lanes 4 and 5**). Thus, the deletion of HABMs from USP17 did not disrupt the catalytic activity of USP17 which is essential for deubiquitination of SDS3 protein. These results suggest that HABMs in USP17 contribute to the interaction between USP17 and SDS3, but do not affect the deubiquitinating activity of USP17 on SDS3.

### HABMs in USP17 and SDS3 Negatively Regulate Cell Proliferation

**Figure 5:**
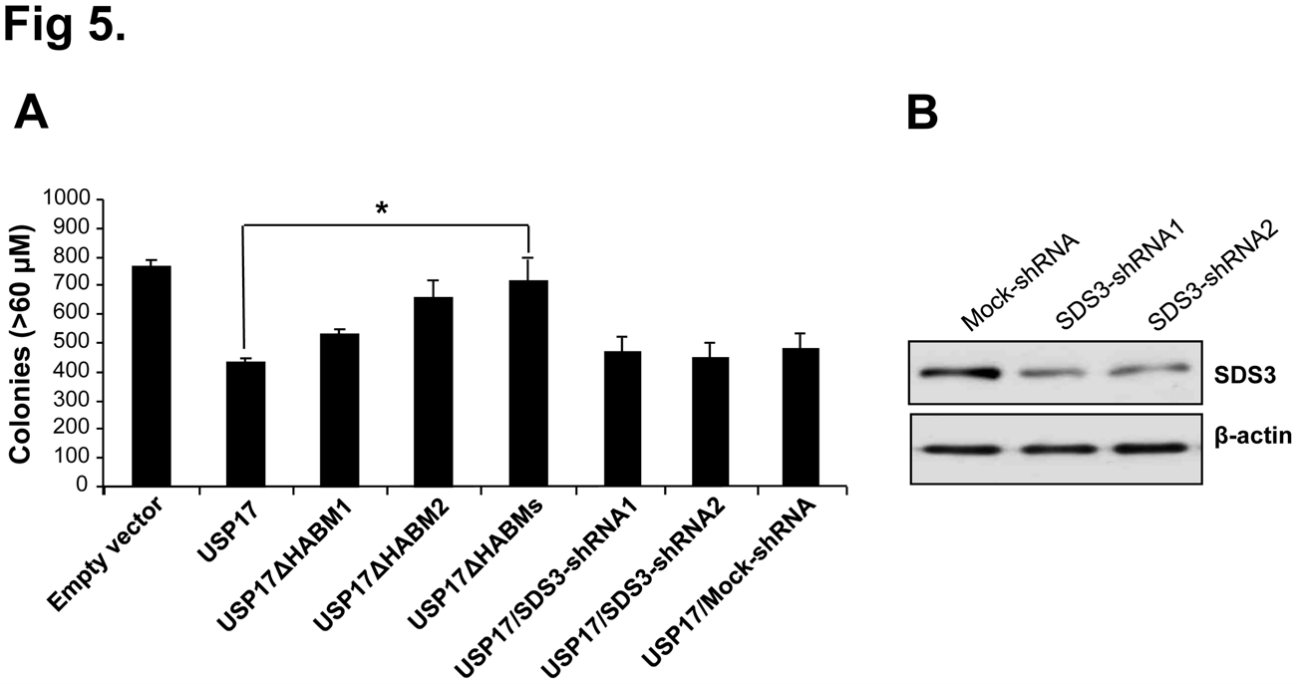
Anti-tumor activity of USP17 and SDS3 containing HABMs. **A**) HeLa cells transfected with empty vector, USP17, USP17ΔHABM1, USP17ΔHABM2, USP17ΔHABMs, USP17/SDS3-shRNA1, USP17/SDS3-shRNA2 and USP17/Mock-shRNA were plated, in triplicate. After 14 days, the colonies were stained and numbers were determined by counting colonies from soft agar assay. A mean of triplicate measurements; bars, Standard deviation, n = 3. *, p<0.05. **B**) The knockdown efficiency of SDS3-shRNA1 and shRNA2 were checked by Western blot analysis in HeLa cells. **C**) HeLa cells transfected with empty vector, SDS3, SDS3ΔHABMs, SDS3/USP17-shRNA1, SDS3/USP17-shRNA2, and SDS3/Mock-shRNA were plated, in triplicate. A mean of triplicate measurements; bars, Standard deviation, n = 3. *, p<0.05. **D**) The knockdown efficiency of USP17-shRNA1 and shRNA2 were checked by Western blot analysis in HeLa cells. **E**) HeLa cells transfected with empty vector, USP17, SDS3, USP17/SDS3, and USP17ΔHABMs/SDS3ΔHABMs were plated, in triplicate. Results represent the average number of colonies formed from three individual experiments. A mean of triplicate measurements; bars, Standard deviation, n = 3. *, p<0.05. **F**) HeLa cells transfected with respective constructs and cell proliferation was assessed by Cell Counting Kit-8 assay. Mean standard deviation of a triplicate experiment is shown. **G**) HeLa cells expressing respective constructs were harvested and stained with annexin V and propidium iodide. The statistical representation of the percentage of apoptosis was recorded. The results represent the mean (n  = 3) of a representative experiment. Error bars represent standard error of the mean. *, p<0.05. **H**) HeLa cells transfected with respective constructs were checked for HABMs-mediated migratory effect of USP17 or SDS3 by wound healing assays. Percentage of migration was statistically analyzed from three separate experiments. n  = 3. *, p<0.05.

We hypothesized that the deletion of HABMs from USP17 showing reduced interaction with SDS3 could be involved in the regulation of cell proliferation. For this purpose, we investigated whether USP17 and SDS3-containing HABMs have an inhibitory effect on anchorage- independent growth of tumor cells. Mock control HeLa cells displayed large number of colonies, while HeLa cells transfected with USP17 alone showed a greater reduction in colony formation (**Figure 5A**). In contrast, USP17ΔHABM1 and USP17ΔHABM2 showed lesser inhibitory effect than USP17. In addition, HeLa cells transfected with USP17 lacking both the HABMs showed a significantly large number and size of colonies which was similar to the mock control. Interestingly, the growth inhibiting effect of USP17 was not altered in the presence of depletion of endogenous SDS3 (**Figure 5A**). Scrambled shRNA (Mock-shRNA) which lacks sequence homology to the genome was used as a control in our studies showed no effect on USP17-mediated cell proliferation (**Figure 5A**). The knockdown efficiency of SDS3-shRNA1 and shRNA2 was analyzed by Western blotting (**Figure 5B**). Similarly, SDS3-transfected cells significantly reduced the size and number of colonies formed on soft agar when compared to the mock control. In contrast, SDS3ΔHABMs-transfected cells showed significant increase in colony formation (**Figure 5C**). Interestingly, the growth inhibiting effect of SDS3 was not changed by the depletion of endogenous USP17 (**Figure 5C**). The knockdown efficiency of USP17-shRNA1 and shRNA2 was checked by Western blot analysis in HeLa cells (**Figure 5D**). Same experiments were repeated in MCF-7 cells and obtained similar results (data not shown).

Further, we demonstrated the inhibitory effect of USP17 and SDS3, and its HABM-deletion constructs on cell growth using soft agar assay. Mock control HeLa cells displayed a large number of colonies, while HeLa cells transfected with USP17 or SDS3 alone showed a greater reduction in colony formation. In addition, USP17 and SDS3 co-transfected cells significantly reduced the size and number of colonies formed on soft agar and exhibited about 75% reduced cell proliferation when compared to a mock control (**Figure 5E**). In contrast, co-transfection of USP17 and SDS3-lacking HABMs showed a significantly lesser inhibitory effect on cell growth. To cross confirm this effect, we performed a cell proliferation assay using a Cell Counting kit-8 for over 72 hrs in three separate experiments. The co-transfection of HABM-deletion construct of USP17 and HABM-deletion construct of SDS3 showed lesser inhibitory effect on cell proliferation when compared with the effect observed in USP17 and SDS3 co-transfected cells (**Figure 5F**). The FACS analysis of the propidium iodide (PI) and annexin V stained cells showed about 5 fold increase in early stage of apoptosis in USP17 and SDS3 co-transfected cells, while cells co-transfected with HABM-deleted USP17 and SDS3 did not show any significant level of apoptosis when compared with a mock control (**Figure 5G**). To further evaluate the effect of HABMs of USP17 and SDS3 on cell migration, wound healing assay was performed. The co-transfection of USP17 and SDS3 showed significant reduction in cell migration when compared with the HABMs deleted USP17 and SDS3 (**Figures 5H**). Thus, the co-transfection of USP17 and SDS3 showed combined effect of tumor growth suppression. Altogether, our result indicates that the growth inhibiting action of USP17 and SDS3 is not mainly regulated by its interaction but due to the presence of HABMs in its sequence.

**Figure 6:**
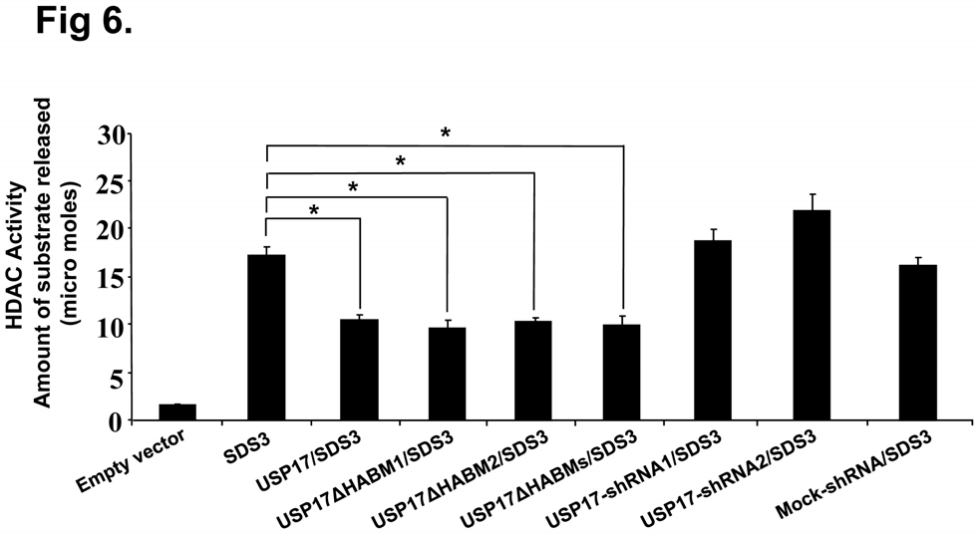
HABMs deleted USP17 does not alter SDS3-associated HDAC activity. HeLa cells were transfected with empty vector, SDS3, USP17/SDS3, USP17ΔHABM1/SDS3, USP17ΔHABM2/SDS3, USP17ΔHABMs/SDS3, USP17-shRNA1/SDS3, USP17-shRNA2/SDS3, and Mock-shRNA/SDS3. HDAC activity on anti-SDS3 immunoprecipitates was determined by measuring released deacetylase substrate using HDAC assay kit. A mean of triplicate measurements; bars, Standard deviation, n = 3. *, p<0.05.

Furthermore, we hypothesized that the HABMs deleted USP17 retaining the deubiquitinating activity on SDS3 is independent of its tumor growth suppressing activity. For this purpose, we investigated HDAC assay on anti-SDS3 immunoprecipitates in three independent experiments. HeLa cells transfected with empty vector, SDS3, USP17/SDS3, USP17ΔHABM1/SDS3, USP17ΔHABM2/SDS3, USP17ΔHABMs/SDS3, USP17-shRNA1/SDS3, USP17-shRNA2/SDS3 and Mock-shRNA/SDS3 were subjected to immunoprecipitation using an anti-SDS3 antibody and HDAC assay was performed. Overexpression of USP17, USP17ΔHABM1, USP17ΔHABM2 and USP17ΔHABMs showed similar SDS3-associated HDAC activity, while the depletion in endogenous expression of USP17 increased the SDS3-associated HDAC activity (**Figure 6**). Thus, our result indicates that deubiquitinating activity of USP17, USP17ΔHABM1, USP17ΔHABM2 and USP17ΔHABMs showed similar regulation on SDS3-associated HDAC activity.

In this study, we identified and characterized HABMs in USP17 and SDS3 showing HA-binding affinity. Furthermore, we provide evidence for a functional interaction between USP17 and SDS3-containing HABMs, which plays a crucial role in regulating cell viability. Deletion of HABMs from USP17 and SDS3 attenuated its inhibitory effect on cell proliferation but could not affect its deubiquitinating activity and its functional regulation on SDS3-associated HDAC activity. Taken together, our data suggest that the HA-binding activity of USP17 may contribute to the growth suppressing activity to balance the action between ubiquitination and deubiquitination to ensure the cellular homeostasis.

## Materials and Methods

### Construction of Expression Vectors

The cDNAs encoding the full length USP17 in pcDNA3.1 expression vector, full length SDS3 in pCS4-Flag expression vector, GST-tagged USP17, His-tagged SDS3, USP17-shRNA1, USP17-shRNA2, SDS3-shRNA1, and SDS3-shRNA2 in pSilencer vectors have been previously described [11]. The word “Mock” in the gene silencing figures refers to a control with a scrambled shRNA sequence. HABM-deletion constructs of USP17 and SDS3 were created by a method for site-directed mutagenesis. USP17ΔHABM1, USP17ΔHABM2, and USP17ΔHABMs were cloned into the pcDNA3.1 vector. SDS3ΔHABMs was subcloned into the pCS4-Flag vector.

### Cell Culture and Transfection

HeLa (human ovarian cancer cell line), MCF-7 cells (human breast adenocarcinoma cell line) and 293T (Human embryonic kidney cell line) cells were grown in DMEM (GIBCO-BRL Rockville, MD, USA) supplemented with 10% FBS and 1% penicillin and streptomycin. Transfections were carried out using polyethyleneimine (Polysciences, Warrington, PA, USA).

### Antibodies

The antibodies used for immunoblotting are polyclonal USP17 antibody (Biomeditech, Seoul, Korea) and anti-SDS3 (ab3740) (Abcam, Cambridge, UK). Anti-myc (9E10) (sc-40), anti-β-actin (sc-47778), anti-His-probe (sc-57598) and anti-GST (sc-53909) antibodies were purchased from Santa Cruz Biotechnology. Anti-Flag (F3165) antibody was purchased from Sigma Aldrich.

### Purification of USP17 and SDS3 Proteins

Bacterial lysate expressing His-tagged SDS3 protein was purified using HisTrap Kit (Amersham Biosciences). Bacterial lysate expressing GST-tagged USP17 was purified using Glutathione-Sepharose beads (GE Healthcare). Purified proteins were subjected to CPC assay.

### HA Binding Assay

HA binding assay was carried out according to the protocol as previously described [13]. Expression constructs such as myc-USP17, myc-USP17ΔHABM1, myc-USP17ΔHABM2, myc-USP17ΔHABMs, Flag-SDS3, and Flag-SDS3ΔHABMs were transiently transfected into 293T cells along with or without different concentrations of HA oligosaccharides using polyethyleneimine (Polysciences, Warrington, PA, USA). In addition, cell lysates were incubated with 20 µg of each USP17 peptide (#1: ATD**RRATQGELK**RD; #2: EQN**KTKPEFNVR**KV) or together in the presence of 50 µg of HA (H5388, Sigma, St. Louis, MO, USA). The samples were loaded onto 7.5% SDS-PAGE gel followed by Western blot analysis.

### Co-immunoprecipitation for Binding and Deubiquitination Assays

HeLa cells were transfected with respective constructs for binding and deubiquitination assays. Co-immunoprecipitation assay was performed as described previously [11]. The antibodies used for immunoblotting are anti-myc, anti-Flag and anti-HA were purchased from Santa Cruz Biotechnology (Santa Cruz, CA, USA).

### Soft Agar Clonogenic Assay

HeLa cells were transfected with expression constructs such as pcDNA3-myc, myc-USP17, myc-USP17ΔHABMs, Flag-SDS3, and Flag-SDS3ΔHABMs. At 48 hrs after transfection, cells were trypsinized and counted. Soft agar assay was performed as previously described [36]. The plates were incubated at 37°C for 10-14 days, and the diameter of tumor colonies were estimated with a microscope equipped with an ocular scale in the eyepiece. Colonies with a diameter greater than 0.2 mm were counted.

### Cell Counting Assay

100 µl of transfected HeLa cell suspension having 50,000-100,000 cells/ml was added to 96-well plate. Cell Counting Kit-8 assay was used and the protocol was followed according to the instructions provided by the company (Dojindo Molecular Technologies, Rockville, MD, USA). Absorbance was measured at 450 nm to determine the cell viability in each well.

### FACS Analysis

HeLa cells expressing USP17, SDS3 and HABMs deleted USP17 or SDS3 constructs were harvested and washed with 1× filtered PBS, and the pellet was resuspended in 100 µl of binding buffer (10 mM Hepes/NaOH (pH 7.4), 140 mM NaCl, 2.5 mM CaCl_2_). 5 µl of annexin V-PE (BD Biosciences) and 10 µl propidium iodide (50 µg/ml, BD Biosciences) were mixed and added to the samples. FACS analysis was performed as described previously [13].

### Wounding Assay

Migration and proliferation rates were assayed by wounding assays. HeLa cells transfected with respective constructs were cultured to near 90% confluence in 100Φ dish. A line was drawn with a marker on the bottom of the dish and cell culture medium was aspirated before wounding. A scratch was made on HeLa monolayer with a sterile 200 µl pipette tip in a definite array. The wounded cell layer was washed twice with PBS and incubated in complete medium and wound closure was documented at 0 h and 16 h. Each analysis was performed in triplicate.

### HDAC Assay

The measurement of HDAC activity was performed using a commercially available kit (BioVision, Mountain View, CA, USA). The procedure was followed as described previously [11]. The samples were measured using Tecan Genios Pro (Tecan, Bubendorf, Switzerland) at 405 nm wavelength.
